# A Sensitive SERS Sensor Combined with Intelligent Variable Selection Models for Detecting Chlorpyrifos Residue in Tea

**DOI:** 10.3390/foods13152363

**Published:** 2024-07-26

**Authors:** Hanhua Yang, Hao Qian, Yi Xu, Xiaodong Zhai, Jiaji Zhu

**Affiliations:** 1 School of Electrical Engineering, Yancheng Institute of Technology, Yancheng 224051, China; 2 College of Ocean Food and Biological Engineering, Jimei University, Xiamen 361021, China; 202361000120@jmu.edu.cn; 3 School of Food and Biological Engineering, Jiangsu University, Zhenjiang 212013, China; zhai_xiaodong@ujs.edu.cn

**Keywords:** surface-enhanced Raman spectroscopy, intelligent variable selection models, chlorpyrifos residue, quantitative analysis, tea

## Abstract

Chlorpyrifos is one of the most widely used broad-spectrum insecticides in agriculture. Given its potential toxicity and residue in food (e.g., tea), establishing a rapid and reliable method for the determination of chlorpyrifos residue is crucial. In this study, a strategy combining surface-enhanced Raman spectroscopy (SERS) and intelligent variable selection models for detecting chlorpyrifos residue in tea was established. First, gold nanostars were fabricated as a SERS sensor for measuring the SERS spectra. Second, the raw SERS spectra were preprocessed to facilitate the quantitative analysis. Third, a partial least squares model and four outstanding intelligent variable selection models, Monte Carlo-based uninformative variable elimination, competitive adaptive reweighted sampling, iteratively retaining informative variables, and variable iterative space shrinkage approach, were developed for detecting chlorpyrifos residue in a comparative study. The repeatability and reproducibility tests demonstrated the excellent stability of the proposed strategy. Furthermore, the sensitivity of the proposed strategy was assessed by estimating limit of detection values of the various models. Finally, two-tailed paired *t*-tests confirmed that the accuracy of the proposed strategy was equivalent to that of gas chromatography–mass spectrometry. Hence, the proposed method provides a promising strategy for detecting chlorpyrifos residue in tea.

## 1. Introduction

Because of its possible health benefits, tea is the most famous and extensively consumed traditional nonalcoholic herbal beverage globally. As natural antioxidants, tea polyphenols play a significant role in preventing and treating cardiovascular disease, cancer, inflammation, and immune disorders [1]. Nevertheless, farmers frequently use different pesticides in tea gardens to protect tea plants from pests, rodents, insects, and weeds, but without following control limits [2]. However, the large-scale use of pesticides can accumulate in tea leaves and easily enter drinkable tea brews, which could raise public health concerns.

Organophosphates (OPs) constitute a group of pesticides specifically designed to control pests, weeds, and plant diseases. The application of OPs is still considered the most effective and accepted method to protect plants, which is crucial for improving agricultural productivity and crop yields [3]. For example, dichlorvos, diazinon, dimethoate, chlorpyrifos, fenitrothion, monocrotophos, malathion, parathion, and quinalphos are commonly used OPs [4]. Among the OPs, chlorpyrifos [*O*,*O*-diethyl *O*-(3,5,6-trichloro-2-pyridinyl)-phosphorothioate] has become one of the most widely available broad-spectrum insecticides since its synthesis by a German chemical scientist in the 1930s for destroying flea beetles, flies, fire ants, maize rootworms, mosquitoes, roundworms, termites, and weeds [5]. Despite the positive effect of chlorpyrifos in modern farming, its residue in the environment can easily cause the pollution of water and soil and harm animals, which in turn poses a health threat to humans through biological accumulation [6]. Numerous reports have demonstrated that chlorpyrifos residue can cause chronic damage to the human body, including gene mutations, endocrine disruption, and neurocognitive impairment [7,8]. Given this, the maximum residue of chlorpyrifos has been regulated by many nations and geographic regions. According to the Commission Implementing Regulation (European Union), the maximum residual limit (MRL) of chlorpyrifos in tea was set to 0.01 mg/kg [9,10]. Hence, it is crucial to construct a sensitive and reliable approach to accurately quantify chlorpyrifos residue to ensure tea safety, thereby protecting consumers’ health and interests.

Abundant routine techniques have been developed for detecting chlorpyrifos residue, such as gas chromatography (GC), high-performance liquid chromatography (HPLC), gas chromatography–mass spectrometry (GC-MS), and liquid chromatography–mass spectrometry (LC-MS) [11,12,13,14]. Despite the fact that these methods present great accuracy and reliability advantages, laborious and complex sample pretreatment, expensive instruments, and skilled operator requirements limit their on-site applications. Consequently, a sensitive, simple, and accurate technique is required to detect trace-level chlorpyrifos residue in tea. 

In the past several years, surface-enhanced Raman spectroscopy (SERS), a hybrid of Raman spectroscopy and nanotechnology, has received considerable attention in food safety, including for the detection of illegal additives, heavy metals, biotoxins, antibiotics, and pesticide residues, due to its high sensitivity, rapidity, and non-destructiveness [15,16,17,18,19]. The SERS signals of analyte molecules can be amplified when adsorbed on or close to the surface of precious metal (such as gold or silver) nano-materials [20,21]. SERS signals can provide rich fingerprint information of analyte molecules, and SERS characteristic peaks are assignable to the vibrational modes of the internal molecular bonding of a compound [22]. In the scientific community, the mainstream view is that chemical and electromagnetic enhancement work together to produce the SERS enhancement effect, while the latter contributes more significantly [23]. Additionally, many studies have revealed that the enhancement factor (EF) was closely related to the SERS sensors’ controllable size and tunable shapes. For example, anisotropic sensors are more effective for generating SERS signals than spherical sensors due to the sharp edges and tips on their surface, which result in abundant “hot spots” [24].

In practical applications, the qualitative or quantitative analysis of SERS spectral data faces many challenges because of its tremendous dimensionality. The main feature of SERS spectral data is that the number of variables is much larger than that of the samples, which leads to the “curse of dimensionality”. The primary risk of modeling such spectral data is over-fitting. To address this problem, partial least squares (PLS) and principal component regression (PCR) have been employed to extract latent variables (LVs) or principal components (PCs) for developing multivariate calibration models, to ultimately realize the goal of dimension reduction and lower the risk of over-fitting. Additionally, many researchers have either theoretically or experimentally demonstrated that introducing variable selection methods into spectroscopic analysis can further improve the performance of qualitative or quantitative analysis models [25,26]. In brief, the advantages of variable selection can be summed up as follows: first, the models’ prediction ability and computational speed can be advanced by removing uninformative or interfering spectral variables; and second, the models’ interpretability can be enhanced by choosing informative spectral variables [27].

**Figure 1:**
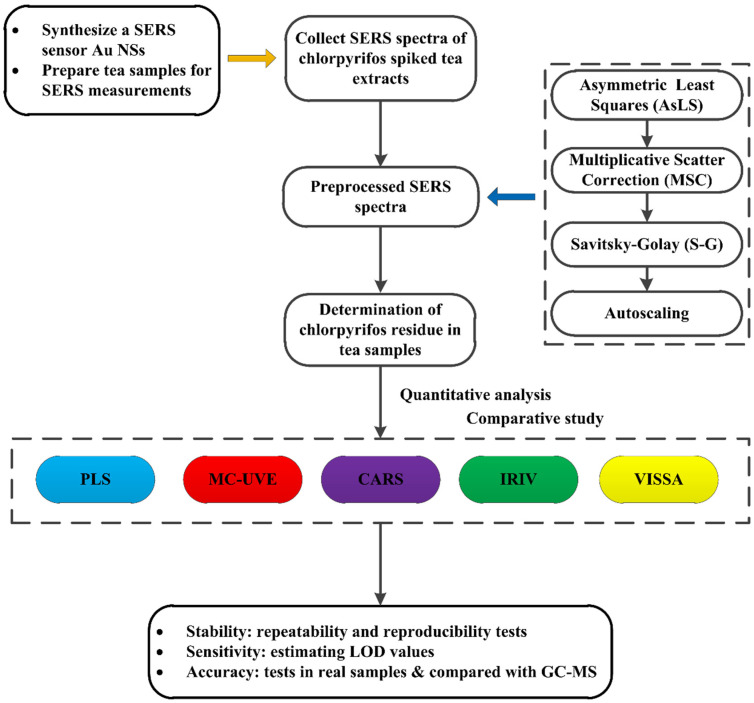
The schematic diagram of the experimental design of this study.

This study aimed to construct an efficient and convenient strategy for detecting trace-level chlorpyrifos residue in tea by employing SERS coupled with intelligent variable selection models. First, a seed-mediated method produced gold nanostars (Au NSs) as the SERS sensor. Second, the SERS spectra of chlorpyrifos-spiked tea extracts were measured by a portable Raman spectrometer. Subsequently, the collected SERS spectra were preprocessed by adopting a spectral data preprocessing strategy. Four intelligent variable selection models, including Monte Carlo-based uninformative variable elimination (MC-UVE), competitive adaptive reweighted sampling (CARS), iteratively retaining informative variables (IRIV), and variable iterative space shrinkage approach (VISSA), were developed. Then, these intelligent variable selection models, along with a PLS model, were used to predict chlorpyrifos residue in tea samples based on the preprocessed SERS spectra. A comparative study of the prediction results obtained by different models was carried out. The proposed strategy’s stability and sensitivity were confirmed by the repeatability and reproducibility tests and the calculated limit of detection (LOD) values. To verify the proposed strategy’s accuracy, the paired *t*-tests were conducted on the detection results yielded by the proposed strategy and those produced by the GC-MS method. In this study, the schematic diagram of the experimental design is shown in Figure 1.

## 2. Materials and Methods

### 2.1. Chemicals and Reagents

A standard sample of chlorpyrifos (C_9_H_11_Cl_3_NO_3_PS, 99.0%) was supplied by Shanghai Pesticide Research Institute Co., Ltd. (Shanghai, China). Chloroauric acid (HAuCl_4_·4H_2_O, 99.9%), trisodium citrate (C_6_H_5_Na_3_O_7_, 99.8%), silver nitrate (AgNO_3_, 99.8%), hydrochloric acid (HCl, 99.9%), ethanol (C_2_H_6_O, 99.8%), ascorbic acid (C_6_H_8_O_6_, 99.9%), methylene chloride (CH_2_Cl_2_, 99.8%), acetonitrile (C_2_H_3_N, 99.8%), ethyl acetate (C_4_H_8_O_2_, 99.8%), anhydrous sodium sulfate (Na_2_SO_4_, 99.9%), and n-hexane (CH_3_(CH_2_)_4_CH_3_, 99.5%) were supplied by Sinopharm Chemical Reagent Co., Ltd. (Shanghai, China). Supelclean ENVI-Carb solid phase extraction (SPE) column (500 mg/6 mL), SIMON Florisil SPE column (500 mg/6 mL), and Agilent HP-5 analytical column (95% dimethyl polysiloxane, 5% diphenyl, 30 m × 0.25 mm × 0.25 μm) were purchased from Shanghai Charm Analytical Technology Co., Ltd. (Shanghai, China). All the reagents were used without further purification.

### 2.2. Instrumentation 

A UV-1800 spectrophotometer (Shimadzu Co., Ltd., Kyoto, Japan) was used to measure ultraviolet–visible (UV–Vis) extinction spectra. Transmission electron microscopy (TEM) using a JEM-2100HR transmission electron microscope (JEOL Co., Ltd., Tokyo, Japan) was used to evaluate the morphology of the SERS sensor. A D8 ADVANCE X-ray diffractometer (Bruker AXS Co., Ltd., Karlsruhe, Germany) was used to examine the SERS sensor’s X-ray diffraction (XRD) patterns. Energy dispersive X-ray spectroscopy (EDS) was used to analyze the elementary composition of the SERS sensor using field emission scanning electron microscopy (Merlin, Carl Zeiss AG, Oberkochen, Germany). The zeta potential of the SERS sensor was measured by a Zetasizer Nano ZEN3600 (Malvern Instruments Co., Ltd., Worcestershire, UK). A GCMS-QP2010 GC-MS (Shimadzu Co., Ltd., Kyoto, Japan) was used to analyze the content of chlorpyrifos in tea samples. A PS-20A ultrasonator (Shenzhen Dekang Technologies Co., Ltd., Shenzhen, China) was used for ultrasonication. A portable Raman spectrometer (RMS1000, Shanghai Oceanhood Opto-Electronics Tech Co., Shanghai, China), equipped with a laser excitation of 785 nm and a fiber optic Raman probe (RPB-785-1.5-FS), was utilized to measure SERS spectra. The key parameters of the portable Raman spectrometer are as follows: the adjustment range of laser power is 5–500 mW; the spectral shift range is 200–3200 cm^−1^; and the spectral resolution is 2 cm^−1^.

### 2.3. Synthesis of Au NSs

First, Au nanoparticles (NPs) were prepared using a reported method [28]. Then, 50 mL of 0.25 mM HAuCl_4_·4H_2_O was placed in a round bottom flask and heated to ~100 °C under stirring. Then, 0.75 mL of 1% C_6_H_5_Na_3_O_7_ was added and refluxed until the solution color changed to wine-red, indicating the successful synthesis of Au NPs. 

Second, following a reported approach [29], a seeded-growth process was applied to synthesize star-like shaped Au NSs based on the prepared Au NPs. First, 250 µL of Au NPs with a diameter of ~17.53 nm was added as a seed solution to 20 mL of 0.25 mM HAuCl_4_·4H_2_O in a 50 mL beaker under stirring at 25 °C. After 5 min, 10 mL of 1 M HCl was added under the same conditions. Then, 0.2 mL of 2 mM AgNO_3_ and 0.1 mL of 0.1 M C_6_H_8_O_6_ were added quickly. After that, the mixture was stirred for ~30 s, and the solution color changed to blue, indicating the successful synthesis of Au NSs. In this study, the synthesized Au NSs served as the SERS sensor. The process diagram of synthesizing Au NSs is shown in Figure S1.

### 2.4. Sample Preparation

Green tea samples were bought at a nearby grocery and stored under refrigerated conditions. For simulating actual samples, each sample of 1 g green tea was homogeneously mixed with 1 mL of chlorpyrifos standard solutions of various concentrations (0.001, 0.01, 0.1, 1, 10, and 100 μg/mL). After the mixtures were dried at ambient temperature, the chlorpyrifos-spiked tea samples with different concentrations (0.001, 0.01, 0.1, 1, 10, and 100 μg/g) were obtained. Afterwards, 10 mL of CH_2_Cl_2_ was mixed with 0.25 g of chlorpyrifos-spiked tea samples in a 25 mL volumetric flask. Then, the mixture was ultrasonicated for 1 h (the power was set to 100 W; and the temperature was set to 30 °C) and filtered through Whatman No.1 filter paper. The process was carried out three times, and the extracts were collected and merged. Subsequently, the extract was concentrated to 1 mL in a rotary evaporator and dried by a nitrogen stream. Finally, the residue was diluted with 1 mL of water containing 5% C_2_H_6_O. After 5 min of centrifuging at 6000 rpm, the supernatant was collected for SERS measurements [30].

Prior to spiking and preparation for SERS measurements, the tea samples were analyzed by GC-MS (a detailed procedure is shown in Supplementary Materials) to confirm that there was no pre-existing chlorpyrifos in the tea samples.

### 2.5. Collection of SERS Spectra and Spectral Preprocessing

First, 100 μL of Au NSs was concentrated 10 times. Then, it was mixed with various concentrations of chlorpyrifos-spiked tea extracts and incubated for 5 min to adsorb chlorpyrifos molecules properly on the surface of Au NSs. Subsequently, 10 μL of the mixture was deposited on a quartz plate for SERS measurements. During SERS measurements, the laser power was adjusted to 100 mW, and the integration time was set to 2 s. Finally, 10 SERS spectra were measured for each concentration, and therefore 60 SERS spectra were acquired in total.

In addition to the chemical information of chlorpyrifos, the raw SERS spectra generally contained multiple data artifacts, for instance, a baseline, instrumental noise, and light scattering effects. To improve the accuracy of the developed models, a spectral data preprocessing approach with four steps was employed [31] to eliminate these data artifacts: (1) asymmetric least squares was adopted for baseline correction; (2) multiplicative scatter correction was used for scatter correction; (3) a Savitsky–Golay smoother was employed for smoothing; and (4) autoscaling was used.

### 2.6. Development of the Partial Least Squares (PLS) Model

PLS is a powerful chemometric tool which can be used for relating two data matrices, ***X*** and ***Y***, by a linear multivariate model, but goes beyond traditional multiple linear regression (MLR) in that it also models the structure of ***X*** and ***Y***. PLS derives its usefulness from its ability to analyze data with many, noisy, strongly collinear, and even incomplete variables in both ***X*** and ***Y*** [32]. In view of the above advantages, PLS has been widely and successfully used for spectral analysis [33,34,35,36,37,38]. This study used PLS to develop a multivariate calibration model for the quantitative analysis of chlorpyrifos content in tea samples. For a spectral matrix ***X*** (*m* × *p*) and a corresponding response matrix ***y*** (*m* × 1), an expression of the relationship between ***X*** and ***y*** in the original space is described as follows:

(1) y=bX+E

where ***b*** denotes the regression coefficient matrix, and ***E*** denotes the residual matrix. Projecting the matrix ***X*** into a latent space, which is a low dimensional subspace:

(2) T=XW

where ***W*** is the projection direction matrix, and ***T*** is a low dimensional representation of ***X***. Therefore, Equation (3) is another expression for Equation (1):

(3) y=TQ+E

where ***Q*** represents the regression coefficients between ***T*** and ***y***.

### 2.7. Development of the Intelligent Variable Selection Models

#### 2.7.1. Development of the MC-UVE Model

Based on the UVE and MC methods, a combination of MC and UVE (MC-UVE) was proposed by Cai et al. for variable selection. This study used MC-UVE to select optimal spectral wavelengths and develop a multivariate calibration model for the quantitative analysis of chlorpyrifos content in tea samples. For a spectral matrix ***X*** (*m* × *p*) and a corresponding response matrix ***y*** (*m* × 1), the main procedures of MC-UVE are described as follows.

Step 1: A MC sampling run was performed *N* times. A portion of samples (e.g., *σ* × *m*) were chosen for every sampling run to generate a new spectra matrix ***X****^i^* and a corresponding matrix ***y****^i^* for developing a PLS model, where *i* = 1, 2, …, *N*.

Step 2: Based on the developed PLS models, a coefficient matrix ***B*** = [***b***_1_, ***b***_2_, …, ***b****p*] was obtained, where ***b****_j_* = [*b*_1*j*_, *b*_2*j*_, …, *b_N_j*]^T^. The stability of each wavelength was calculated as follows:

(4) $$s_{j}=mean(b_{j})/std(b_{j})\;j=1,2,\ldots ,p$$

where mean(***b****_j_*) and std(***b****_j_*) denote the mean and standard deviation of the regression coefficients of wavelength *j*, respectively.

Step 3: Based on the generated stability, a few (e.g., *M*) of the informative (stable) wavelengths were chosen for the development of the final PLS model. All the wavelengths were ranked in descending order according to their stability, and the stability of the *M*th wavelength was set as the cutoff value. If the stability of the wavelength was less than the cutoff value, it was eliminated; otherwise, it was retained. Finally, the optimal spectral wavelengths were obtained.

**Figure 2:**
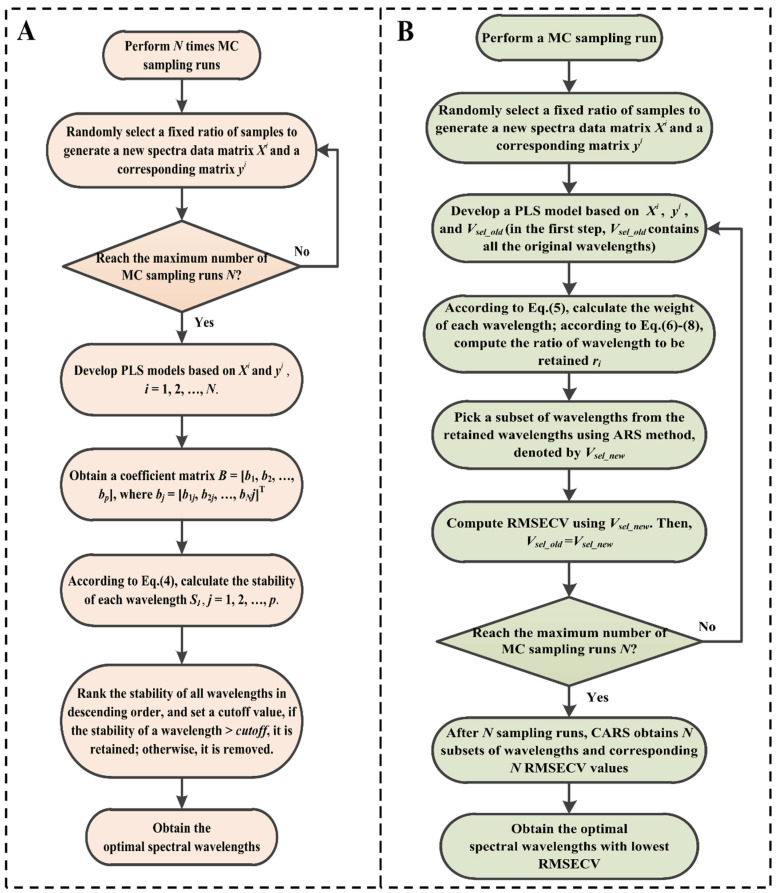
(**A**) A flowchart of MC-UVE. (**B**) A flowchart of CARS.

Referring to previous literature [39], the number of MC sampling runs *N* was set to 100, and the ratio *σ* was set to 0.8. A flowchart of MC-UVE is shown in Figure 2A.

#### 2.7.2. Development of the CARS Model

Based on Darwin’s evolution theory, Li et al. proposed a variable selection method termed CARS. CARS realizes high computational efficiency by introducing MC and exponentially decreasing function (EDF) strategies and can prevent combination explosion in variable selection. This study employed CARS to select optimal spectral wavelengths and develop a multivariate calibration model for quantitative analysis of chlorpyrifos content in tea samples. For a spectral matrix ***X*** (*m* × *p*) and a corresponding response matrix ***y*** (*m* × 1), the main procedures of CARS are described as follows.

Step 1: A MC sampling run was performed. In this sampling run, a portion of samples (e.g., *σ* × *m*) were chosen to generate a new spectral matrix ***X***′ and a corresponding matrix ***y***′ for developing a PLS model.

Step 2: For the PLS model built by ***X***′ and ***y***′, a coefficient vector ***b*** = [*b*_1_, *b*_2_, …, *b_p_*]^T^ was obtained. To evaluate each wavelength’s importance, a normalized weight for each wavelength was defined as follows:

(5) $$w_{j}=\frac{\left|b_{j}\right|}{\sum_{j=1}^{p}\left|b_{j}\right|}\;j=1,2,\ldots ,p$$

Step 3: The wavelengths with comparatively small weights were eliminated by the EDF strategy. In the *i*th sampling run, the ratio of wavelengths retained was calculated as follows:

(6) $$r_{i}=ae^{-ki}$$

where *a* and *k* are two constants depending on two conditions: (1) in the first sampling run, all wavelengths were used for developing a PLS model, so *r*_1_ = 1; (2) in the *N*th sampling run, there were only two wavelengths retained, so *r_N_* = 2/*p*. Then, *a* and *k* could be computed as follows:

(7) $$a={\left(\frac{p}{2}\right)}^{1/\left(N-1\right)}$$

(8) $$k=\frac{\ln\left(p/2\right)}{N-1}$$

where *ln* represents the logarithm (base e). After EDF-based enforced wavelength selection, a new wavelength subset was generated via the adaptively reweighted sampling (ARS) method for developing a PLS model, and the corresponding five-fold root mean squared error of cross-validation (RMSECV) value was calculated.

Step 4: After performing *N* times MC sampling runs, *N* wavelength subsets and the corresponding *N* RMSECV values were obtained. The wavelength subset with the lowest RMSECV value was chosen as the ideal spectral wavelengths.

Referring to previous literature [40], the number of MC sampling runs *N* was set to 500, and the ratio *σ* was set to 0.8. A flowchart of CARS is shown in Figure 2B.

#### 2.7.3. Development of the IRIV Model

Based on model population analysis (MPA), Yun et al. proposed an effective variable selection method called IRIV. IRIV classifies variables into four categories: strongly informative, weakly informative, uninformative, and interfering variables. During iterations, both the strongly and weakly informative variables are kept, and the variables that are uninformative and interfering are removed. Ultimately, a backward elimination strategy is carried out on the retained variables to obtain the optimal variables. This study employed IRIV to select the optimal spectral wavelengths and develop a multivariate calibration model for the quantitative analysis of chlorpyrifos content in tea samples. For a spectral matrix ***X*** (*m* × *p*) and a corresponding response matrix ***y*** (*m* × 1), the main procedures of IRIV are described as follows.

Step 1: A binary matrix ***M*** (*N* × *p*) was generated for sampling, where *N* denotes the number of sampling runs. In ***M***, half of the elements in each column were set to “1”, and the other half were set to “0”, indicating that each wavelength’s sampling weight was the same. Herein, “1” represents that the corresponding wavelength was selected, while “0” means that the corresponding wavelength was not selected. A new binary matrix ***M***′ was obtained by permutating elements in each column, and its dimension was the same as that of ***M***. The schematic diagram of this step is shown in Figure S2.

Step 2: A PLS model was developed for each row in ***M***′, and the corresponding five-fold RMSECV was calculated. Hence, it was possible to obtain a RMSECV vector (*N* × 1), which was represented as RMSECV_0_.

Step 3: A novel strategy was introduced to evaluate the importance of each wavelength. For the *i*th (*i* = 1,2, …, *p*) wavelength, a matrix ***M***1′ was generated by changing all the “1” elements in the *i*th column of ***M***′ to “0” and all the “0” elements in the *i*th column to “1”, while keeping other columns of ***M***′ unchanged. Based on ***M***1′, RMSECV*_i_* (*N* × 1) was obtained. Then, *Φ*_0_ and *Φ_i_* were employed to evaluate each wavelength’s importance using Equation (9):

(9) $$\Phi_{0k}=\left\{\begin{array}{c} kth\;{RMSECV}_{0}\;if\;M_{ki}^{\prime }=1\\ kth\;{RMSECV}_{i}\;if\;{M1}_{ki}^{\prime }=1 \end{array}\right.,\;\Phi_{ik}=\left\{\begin{array}{c} kth\;{RMSECV}_{0}\;if\;M_{ki}^{\prime }=0\\ kth\;{RMSECV}_{i}\;if\;{M1}_{ki}^{\prime }=0 \end{array}\right.$$

where $M_{ki}^{\prime }$ represents the value in the *k*th row and *i*th column of ***M***′, and ${M1}_{ki}^{\prime }$ represents the value in the *k*th row and *i*th column of ***M***1′. 

Step 4: The mean value of *Φ*_0_ and *Φ_i_* were calculated, which were denoted as MEAN*_i_*_,include_ and MEAN*_i_*_,exclude_, respectively. It was possible to compute the difference between the two mean values as follows:

(10) $${DM}_{i}={MEAN}_{i,include}-{MEAN}_{i,exclude}$$

By using Equation (10) and the Mann–Whitney *U* test (*p* = 0.05), the wavelengths were readily divided into the following four groups:

(11) $$\left\{\begin{array}{c} strongly\;informative\;if\;{DM}_{i}<0,\;P_{i}<0.05\\ weakly\;informative\;if\;{DM}_{i}<0,\;P_{i}>0.05\\ uninformative\;if\;{DM}_{i}>0,\;P_{i}>0.05\\ interfering\;if\;{DM}_{i}>0,\;P_{i}<0.05 \end{array}\right.$$

Step 5: The strongly and weakly informative wavelengths were retained in place after eliminating the uninformative and interfering wavelengths. Then, the next round was performed by returning to Step 1 until no uninformative or interfering wavelengths remained.

Step 6: A backward elimination strategy was carried out on the retained wavelengths to obtain the optimal wavelengths.

**Figure 3:**
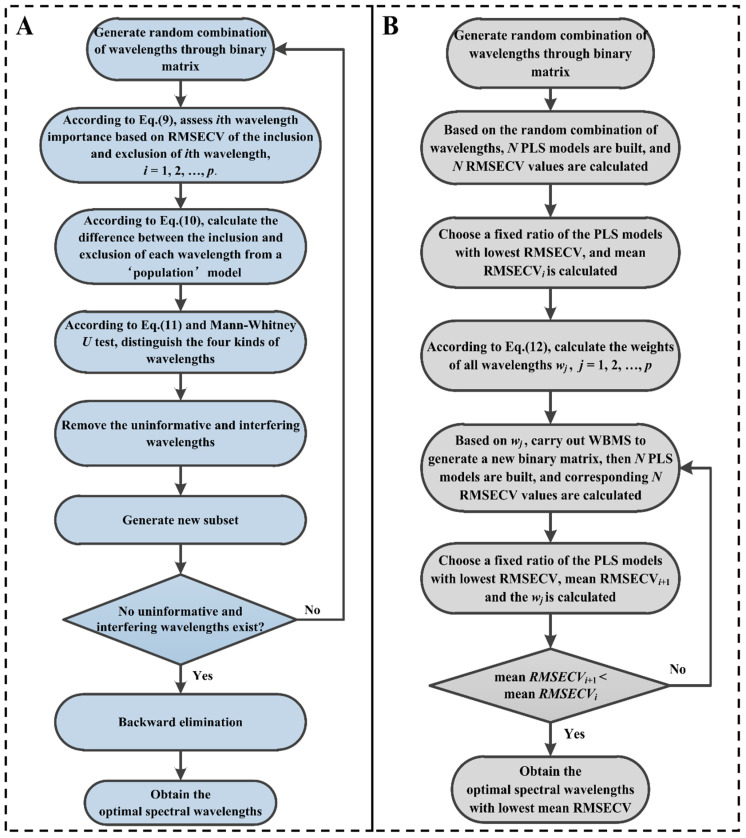
(**A**) A flowchart of IRIV. (**B**) A flowchart of VISSA.

Referring to previous literature [41], the number of binary matrix sampling runs *N* was set to 500. A flowchart of IRIV is shown in Figure 3A.

#### 2.7.4. Development of the VISSA Model

Combining MPA and weighted binary matrix sampling (WBMS), Deng et al. proposed a wavelength selection approach termed VISSA. In contrast to most current variable selection techniques, VISSA assesses space performance statically at every stage of the optimization process. Furthermore, the performance of the new variable space is better than that of the previous one during optimization. This study employed VISSA to select optimal spectral wavelengths and develop a multivariate calibration model for the quantitative analysis of chlorpyrifos content in tea samples. For a spectra matrix ***X*** (*m* × *p*) and a corresponding response matrix ***y*** (*m* × 1), the main procedures of VISSA are illustrated as follows:

Step 1: A binary matrix ***M*** (*N* × *p*) was generated for sampling, where *N* is the number of sampling runs. In ***M***, half of the elements in each column were set to “1”, and the other half were set to “0”, and therefore each wavelength’s sampling weight was initially set to 0.5. Herein, “1” represents that the corresponding wavelength was selected, while “0” means that the corresponding wavelength was not selected. By permutating elements in each column, a new binary matrix ***M***′ was obtained, and its dimension was the same as that of ***M***. Based on each row in ***M*′**, *N* PLS models were built, and *N* RMSECV values were calculated. Figure S3 displays the schematic representation for this step.

Step 2: A portion of the developed PLS models (e.g., *σ* × *N*) that had the lowest RMSECV values were chosen. The mean RMSECV value for those chosen models was calculated and recorded as ${\bar{RMSECV}}_{i}$, where *i* represents the current optimization step. The weights for all wavelengths could be calculated as follows:

(12) $$w_{j}=\frac{f_{j}}{\sigma \times N}\;j=1,2,\ldots ,p$$

where *f_j_* represents the frequency of wavelength *j* in the chosen *σ* × *N* models, and *w_j_* denotes the weight of wavelength *j* (0 ≤ *w_j_* ≤ 1).

Step 3: WBMS was executed using the wavelength weights that were previously acquired to generate a new binary matrix. Then, *N* PLS models were built based on the new binary matrix. Referring to the method described in Step 2, the mean RMSECV value was calculated and recorded as ${\bar{RMSECV}}_{i+1}$. The weights for all wavelengths were also recalculated by referring to Equation (12). 

Step 4: ${\bar{RMSECV}}_{i}$ and ${\bar{RMSECV}}_{i+1}$ were compared. If ${\bar{RMSECV}}_{i+1}$ < ${\bar{RMSECV}}_{i}$, Steps 3 and 4 were performed until the mean RMSECV value of the updated models exhibited no additional improvement. Finally, the optimal spectral wavelengths were obtained.

Referring to previous literature [42], the number of binary matrix sampling runs *N* was set to 1000, and the ratio *σ* was set to 0.05. A flowchart of VISSA is shown in Figure 3B.

### 2.8. Model Evaluation Metrics

The prediction performance of these models was assessed by the root mean squared error of the calibration set (RMSEC), the root mean squared error of the prediction set (RMSEP), the coefficient of determination of the calibration set ($R_{C}^{2}$), the coefficient of determination of the prediction set ($R_{P}^{2}$), and the ratio of performance to deviation (RPD). In this study, all algorithms and calculations were performed by using MATLAB R2020a. 

## 3. Results and Discussion

### 3.1. Characterization of Au NSs

**Figure 4:**
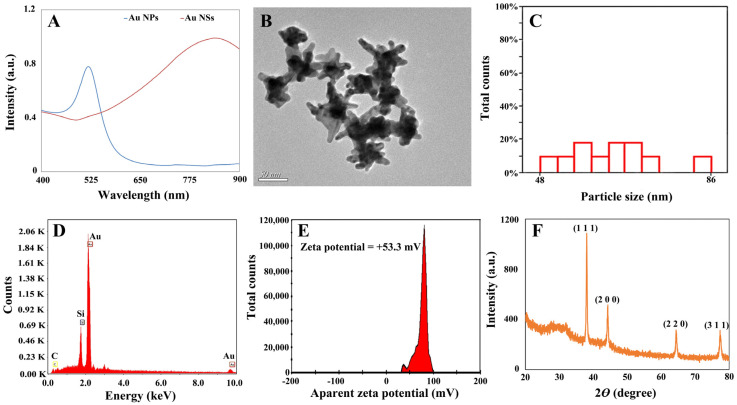
(**A**) The UV–Vis extinction spectrum of Au NSs. (**B**) The TEM image of Au NSs. (**C**) The statistical histogram of the particle size of Au NSs. (**D**) The EDS of Au NSs. (**E**) The zeta potential of Au NSs. (**F**) The XRD pattern of Au NSs.

As displayed in Figure 4A, the UV–Vis extinction spectrum of Au NSs showed a broad peak at 867 nm, whereas the UV–Vis extinction spectrum of Au NPs (~17.53 nm, shown in Figure S4) showed a narrow peak at 527 nm, the same as that reported in previous literature [43,44]. The morphology of the Au NSs was validated by a TEM image (shown in Figure 4B). The size of Au NSs was ~64.09 nm, with an average of eight branches. The statistical histogram of the particle size of Au NSs is shown in Figure 4C. The signal of gold in the EDS histogram revealed that gold was the main element of the synthesized Au NSs, as shown in Figure 4D. Furthermore, the presence of the silicon signal was due to the use of a silicon wafer as the EDS sample template, and the carbon signal was from the reducing agents. As shown in Figure 4E, the surface charge of the Au NSs was positive, with the zeta potential value of +53.3 mV. The XRD pattern of Au NSs exhibited peaks at 38.04°, 44.43°, 64.70°, and 77.38°, and these peaks were related to the crystalline planes of the face-centered-cubic of (111), (200), (220), and (311), respectively, indicative of the crystallinity of Au NSs (JCPDS no.99-0056), as shown in Figure 4F. Additionally, the enhancement factor (EF) of Au NSs was estimated to be 1.06 × 10^5^ (a detailed calculation is shown in Supplementary Materials), which demonstrated that it was capable of measuring the SERS spectra of chlorpyrifos residue in tea based on Au NSs.

### 3.2. Analysis of the Collected SERS Spectra

**Figure 5:**
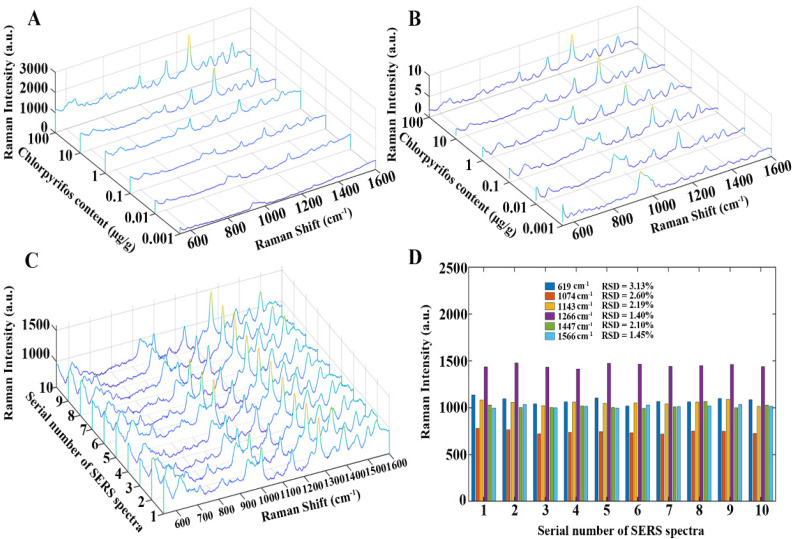
(**A**) Six raw SERS spectra of chlorpyrifos-spiked tea extracts (concentrations: 0.001–100 μg/g). (**B**) Six raw SERS spectra preprocessed by the proposed spectral preprocessing strategy. (**C**) Ten raw SERS spectra of chlorpyrifos-spiked tea extracts (concentration: 1 μg/g). (**D**) The RSD values of the Raman intensities at characteristic peaks: 619, 1074, 1143, 1266, 1447, and 1566 cm^−1^ for the 10 raw SERS spectra (concentration: 1 μg/g).

The SERS spectra of chlorpyrifos-spiked tea extracts were measured using Au NSs. Figure 5A shows six raw SERS spectra of chlorpyrifos-spiked tea extracts over the 0.001–100 μg/g concentration range. According to the theoretical molecular vibrations of chlorpyrifos calculated by the density functional theory (DFT) in a previous report [45], six primary characteristic peaks found at 619, 1074, 1143, 1266, 1447, and 1566 cm^−1^ were assigned to P=S stretching, P-O-C stretching, C-H wagging, ring stretching, C-H wagging, and C=C stretching, respectively. From the perspective of the molecular structure of chlorpyrifos, an internal P=S double bond is responsible for its strong adsorption to metal ions, forming new covalent bonds such as Au-S, Ag-S, and Cu-S [46]. The high affinity of chlorpyrifos toward metal ions facilitated its Raman signal amplification. The proposed spectral preprocessing approach was then applied to the six raw SERS spectra, and the resultant SERS spectra are shown in Figure 5B. The preprocessed SERS spectra were smoother, and the baseline interference was removed, both of which were advantageous for subsequent quantitative analysis. Figure 5C shows 10 raw SERS spectra of chlorpyrifos-spiked tea extracts with a concentration of 1 μg/g, and the relative standard deviation (RSD) values of the Raman intensities at six characteristic peaks were computed. As shown in Figure 5D, the computed RSD values were all less than 5.0%, which implied the high consistency of the measured SERS spectra and the excellent repeatability of the Au NSs.

### 3.3. Detection of Chlorpyrifos Residue in Tea Samples Via Different Models

To predict chlorpyrifos residue in tea samples, five models, PLS, MC-UVE, CARS, IRIV, and VISSA, were applied to the SERS spectra that had been preprocessed. Before developing these models, the preprocessed SERS spectra dataset was partitioned into a calibration set (six spectra were chosen at random for each concentration, 36 spectra in total) and a prediction set (the remaining four spectra for each concentration, 24 spectra in total). Additionally, the values in the response matrix ***y*** (i.e., the chlorpyrifos content) were logarithmically preprocessed. The performance of various models was assessed by the model evaluation metrics. The prediction results yielded by different models are tabulated in Table 1.

#### 3.3.1. Results of the PLS Model

**Figure 6:**
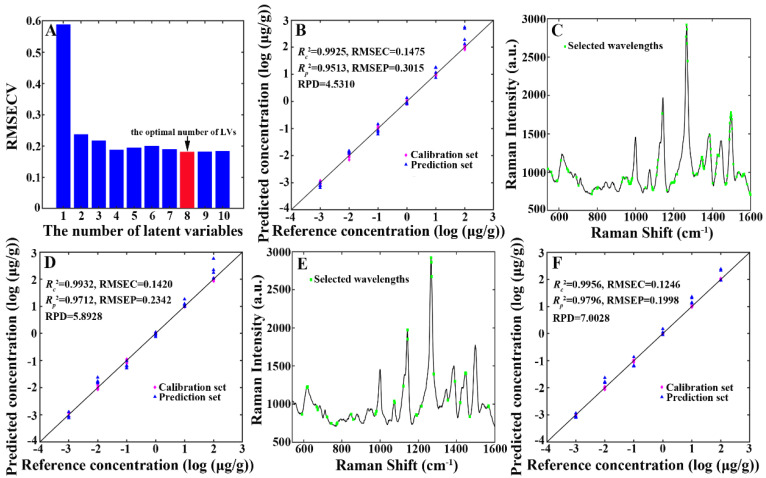
(**A**) The change in RMSECV values during the cross-validation procedure in the PLS model. (**B**) A scatter plot illustrating the correlation between the reference and the PLS model’s predicted chlorpyrifos residue concentration. (**C**) The wavelengths selected by the MC-UVE model. (**D**) A scatter plot illustrating the correlation between the reference and the MC-UVE model’s predicted chlorpyrifos residue concentration. (**E**) The wavelengths selected by the IRIV model. (**F**) A scatter plot illustrating the correlation between the reference and the IRIV model’s predicted chlorpyrifos residue concentration.

PLS was used to develop a multivariate calibration model, which was then used for predicting chlorpyrifos residue concentration in tea samples. As shown in Table 1, the established PLS model generated the subsequent outcomes: the optimal number of LVs (nLVs) = 8, RMSEC = 0.1475, $R_{c}^{2}$ = 0.9925, RMSEP = 0.3015, $R_{p}^{2}$ = 0.9513, and RPD = 4.5310. The change in RMSECV values during the cross-validation procedure is displayed in Figure 6A. A scatter plot illustrating the correlation between the reference and the PLS model’s predicted chlorpyrifos residue concentration is presented in Figure 6B.

#### 3.3.2. Results of the MC-UVE Model

MC-UVE was used to choose the valuable wavelengths from the SERS spectra and develop a multivariate calibration model, which was then used to predict chlorpyrifos residue concentration in tea samples. As shown in Table 1, the developed MC-UVE model generated the following results: nLVs = 10, the number of selected wavelengths (nWAV) = 100, RMSEC = 0.1420, $R_{c}^{2}$ = 0.9932, RMSEP = 0.2342, $R_{p}^{2}$ = 0.9712, and RPD = 5.8928. Figure 6C shows the wavelengths chosen by the MC-UVE model. Of these 100 chosen wavelengths, the wavelengths at 1056, 1139, 1264, 1266, 1268, 1269, 1271, 1560, 1562, 1564, and 1566 cm^−1^ were in the neighborhood of the characteristic peaks of chlorpyrifos: 1074, 1143, 1266, and 1566 cm^−1^. A scatter plot illustrating the correlation between the reference and the MC-UVE model’s predicted chlorpyrifos residue concentration is presented in Figure 6D.

#### 3.3.3. Results of the IRIV Model

IRIV was employed to select the valuable wavelengths from the SERS spectra and develop a multivariate calibration model, which was then used to predict chlorpyrifos residue concentration in tea samples. As shown in Table 1, the built IRIV model yielded the following results: nLVs = 7, nWAV = 27, RMSEC = 0.1246, $R_{c}^{2}$ = 0.9956, RMSEP = 0.1998, $R_{p}^{2}$ = 0.9796, and RPD = 7.0028. Figure 6E shows the wavelengths chosen by the IRIV model. Among these 27 chosen wavelengths, the wavelengths at 617, 619, 1073, 1075, 1143, 1145, 1266, 1268, 1269, 1445, 1447, 1449, 1566, and 1568 cm^−1^ were in the neighborhood of the characteristic peaks of chlorpyrifos: 619, 1074, 1143, 1266, 1447, and 1566 cm^−1^. A scatter plot illustrating the correlation between the reference and the IRIV model’s predicted chlorpyrifos residue concentration is presented in Figure 6F.

#### 3.3.4. Results of the CARS Model

**Figure 7:**
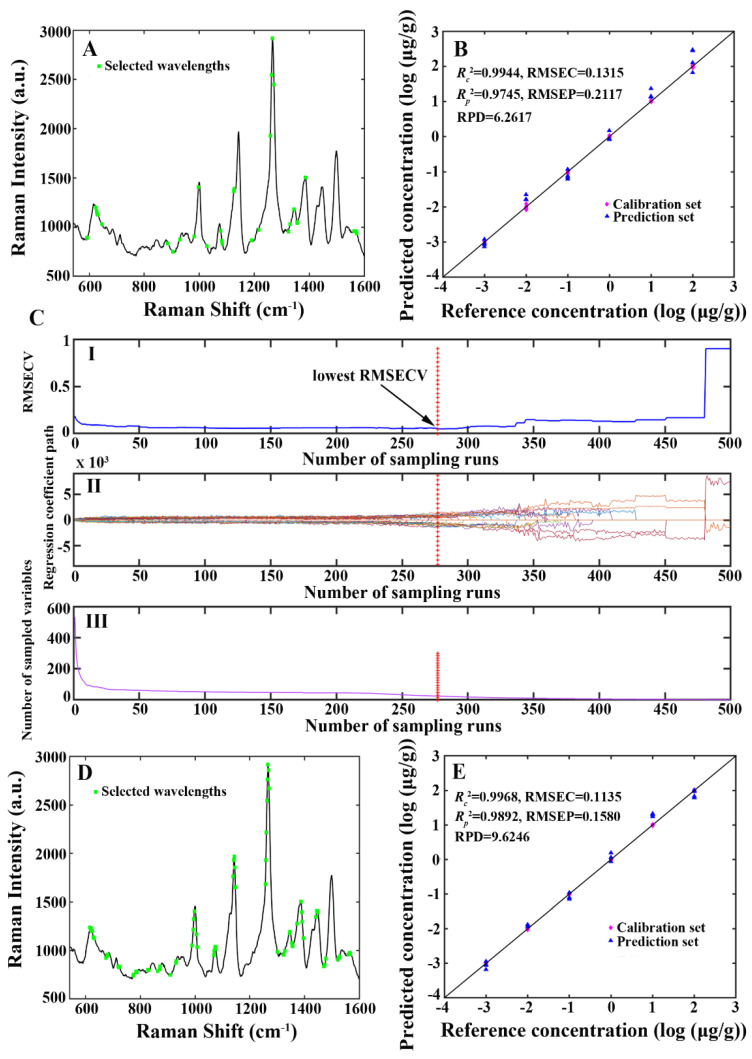
(**A**) The wavelengths selected by the CARS model. (**B**) A scatter plot illustrating the correlation between the reference and the CARS model’s predicted chlorpyrifos residue concentration. (**C**-**I**) The change in RMSECV values for various MC sampling runs. (**C**-**II**) The regression coefficient path for various MC sampling runs. (**C**-**III**) The number of sampled variables for various sampling runs. (**D**) The wavelengths selected by the VISSA model. (**E**) A scatter plot illustrating the correlation between the reference and the VISSA model’s predicted chlorpyrifos residue concentration.

CARS was adopted to select the valuable wavelengths from the SERS spectra and develop a multivariate calibration model, which was then used to predict chlorpyrifos residue concentration in tea samples. As shown in Table 1, the developed CARS model obtained the subsequent outcomes: nLVs = 9, nWAV = 23, RMSEC = 0.1315, $R_{c}^{2}$ = 0.9944, RMSEP = 0.2117, $R_{p}^{2}$ = 0.9745, and RPD = 6.2617. Figure 7A shows the wavelengths chosen by the CARS model. Of these 23 chosen wavelengths, the wavelengths at 624, 626, 628, 1077, 1081, 1258, 1262, 1266, 1271, 1562, 1569, and 1572 cm^−1^ were in the neighborhood of the characteristic peaks of chlorpyrifos: 619, 1074, 1266, and 1566 cm^−1^. A scatter plot illustrating the correlation between the reference and the CARS model’s predicted chlorpyrifos residue concentration is presented in Figure 7B. Figure 7C-I displays the change in RMSECV values for various MC sampling runs. The RMSECV values decreased during sampling runs 1 to 277 as the uninformative or interfering wavelengths were eliminated. Subsequently, the RMSECV values increased during sampling runs 290 to 500 due to the informative wavelengths being removed. Figure 7C-II and Figure 7C-III display the regression coefficient path and the number of sampled variables corresponding to various sampling runs. It can be seen from Figure 7C-III that the progress of variable selection of CARS was split into two steps: first, the wavelengths were eliminated rapidly, which implied a “fast selection” phase; second, the wavelengths were eliminated gradually, which signified a “refined selection” phase. 

#### 3.3.5. Results of the VISSA Model

VISSA was used to select the valuable wavelengths from the SERS spectra and develop a multivariate calibration model, which was then used to predict chlorpyrifos residue concentration in tea samples. As shown in Table 1, the constructed VISSA model obtained the following results: nLVs = 10, nWAV = 78, RMSEC = 0.1135, $R_{c}^{2}$ = 0.9968, RMSEP = 0.1580, $R_{p}^{2}$ = 0.9892, and RPD = 9.6246. Figure 7D shows the wavelengths chosen by the VISSA model. Among these 78 selected wavelengths, the wavelengths at 615, 617, 619, 622, 624, 1067, 1069, 1071, 1073, 1075, 1141, 1143, 1145, 1147, 1264, 1266, 1268, 1269, 1443,1445, 1447, 1449, 1562, 1564, 1566, and 1568 cm^−1^ were in the neighborhood of the characteristic peaks of chlorpyrifos: 619, 1074, 1143, 1266, 1447, and 1566 cm^−1^. A scatter plot illustrating the correlation between the reference and the VISSA model’s predicted chlorpyrifos residue concentration is presented in Figure 7E.

### 3.4. Comparison of Various Models

The RPD value of each variable selection model was higher than 5.0 (Table 1), which signified that all models effectively predicted chlorpyrifos residue concentration in tea samples. Nevertheless, thoroughly examining the prediction performance of the various models remains valuable. Herein, the PLS model was regarded as a benchmark, and the prediction performance of MC-UVE, CARS, IRIV, and VISSA improved in comparison. For the MC-UVE, CARS, IRIV, and VISSA models: (1) the RMSEC values decreased by 3.7%, 10.8%, 15.5%, and 23.1%, respectively; (2) the $R_{c}^{2}$ increased by 0.1%, 0.2%, 0.3%, and 0.4%, respectively; (3) the RMSEP values decreased by 22.3%, 29.8%, 33.7%, and 47.6%, respectively; (4) the $R_{p}^{2}$ values increased by 2.1%, 2.4%, 3.0%, and 4.0%, respectively; and (5) the RPD values increased by 30.1%, 38.2%, 54.6%, and 112.4%, respectively.

In contrast, the prediction ability of the PLS model was inadequate, which was because all wavelengths were included in the modeling. The MC-UVE and CARS models improved the prediction ability compared to the PLS model by selecting informative wavelengths. The outcomes of the IRIV model were superior to those of the MC-UVE and CARS models, which was also validated by the distribution of the selected wavelengths, as they were more concentrated near the characteristic peaks of chlorpyrifos. Nevertheless, the IRIV model considered wavelength importance separately and only partially used wavelength combination information. Hence, it was difficult for the IRIV model to select the optimal wavelength combination. The VISSA model outperformed the other four models, which mainly benefited from the following three aspects: (1) a soft space shrinkage strategy, (2) the optimization of variable space, and (3) the effect of variable combination.

(1) Soft space shrinkage strategy

Backward elimination and EDF are hard space shrinkage approaches commonly used in most variable selection methods. Conversely, VISSA offers a more sophisticated variable space shrinkage method known as a soft space shrinkage technique. Compared with the hard space shrinkage approaches, the soft space shrinkage method requires more time to eliminate the variables in each step when there are many variables, but the risk of eliminating essential variables is lower.

(2) Optimization of variable space

Decisions on whether variables will be included or removed should be made repeatedly during variable optimization. On-time modeling increases the risk of making poor decisions, such as removing essential variables or choosing uninformative ones, and therefore statistical decisions are required. Moreover, when the variable space shrinks, the newly generated space should perform better than the previous one, which is reflected in each round of VISSA; thus, a decrease in the mean value of RMSECV of sub-models is guaranteed.

(3) Effect of variable combination

The variable combination information is a critical factor that various variable selection methods should be considered. This is because some variables do not significantly influence the model initially; however, when coupled with other variables, they play a significant role in modeling. Unlike other models that directly eliminate this type of variable, less important variables are given small weights by VISSA and are permitted to remain in the model for additional analysis. This process is demonstrated by the change in the weights of each wavelength during the iterations (shown in Figure S5).

In brief, an unambiguous ranking for the various models concerning prediction performance can be summarized as: VISSA > IRIV > CARS > MC-UVE > PLS.

### 3.5. Stability and Sensitivity of the Proposed Method

**Figure 8:**
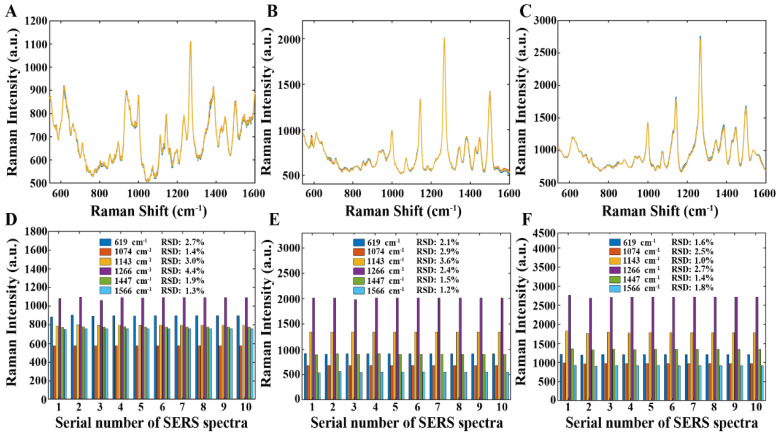
The raw SERS spectra of chlorpyrifos-spiked tea extracts with concentrations: (**A**) 0.1 μg/g, (**B**) 10 μg/g, and (**C**) 100 μg/g. The RSD values of Raman intensities at six characteristic peaks (619, 1074, 1143, 1266, 1447, and 1566 cm^−1^) corresponding to concentrations: (**D**) 0.1 μg/g, (**E**) 10 μg/g, and (**F**) 100 μg/g.

**Figure 9:**
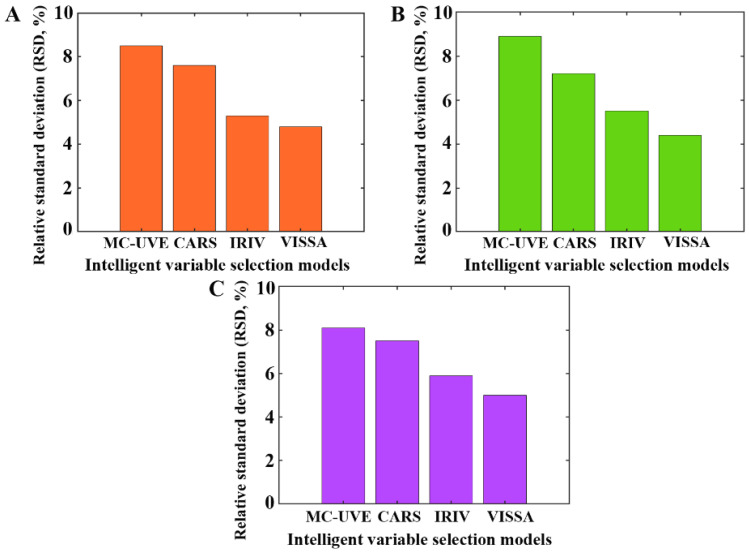
The RSD values generated by various models applied on the SERS spectra of chlorpyrifos-spiked tea extracts with different concentrations: (**A**) 0.1 μg/g, (**B**) 10 μg/g, and (**C**) 100 μg/g.

In this study, the stability of the proposed method was analyzed regarding two aspects: (1) the synthesized SERS sensor Au NSs and (2) the developed intelligent variable selection models. The repeatability of the Au NSs was evaluated in Section 3.2, and a satisfactory result was obtained. In this section, the reproducibility of the Au NSs was assessed by remeasuring the SERS spectra of chlorpyrifos-spiked tea extracts with three distinct concentrations (0.1, 10, and 100 μg/g) on three consecutive days, and 10 SERS spectra were collected for each concentration. Then, the RSD values of the Raman intensities at six characteristic peaks (619, 1074, 1143, 1266, 1447, and 1566 cm^−1^) were computed. As shown in Figure 8, all the computed RSD values were below 5.0%. Subsequently, the remeasured SERS spectra underwent spectral preprocessing and then were fed into the developed models for predicting chlorpyrifos residue concentration. Herein, 30 replicate runs of each intelligent variable selection model were performed for prediction, and then the RSD values of the RMSEP values were recorded for evaluating the stability of the developed intelligent variable selection models. As shown in Figure 9, the RSD values generated by various models on these SERS spectra were all less than 10.0%. Hence, the above results validated that the proposed strategy was stable, and the stability of VISSA model was superior than that of other intelligent variable selection models.

LOD is a crucial metric that was used to assess the sensitivity of the proposed strategy. Based on the mean relative error (MRE) evolution method proposed by Oleneva et al. (detailed in Supplementary Materials) [47], the estimated LOD values of the various models were as follows: LOD_MC-UVE_ = 5.6 × 10^−4^ μg/g, LOD_CARS_ = 5.1 × 10^−4^ μg/g, LOD_IRIV_ = 4.5 × 10^−4^ μg/g, and LOD_VISSA_ = 3.9 × 10^−4^ μg/g. The MRL of chlorpyrifos in tea set by the European Union is 0.01 mg/kg (1.0 × 10^−2^ μg/g). The estimated LOD values of all the models were lower than the MRL value. Hence, the sensitivity of the proposed method is qualified to determine chlorpyrifos residue in tea samples accurately.

### 3.6. Determination of Chlorpyrifos Content in Real Samples

A batch of green tea samples containing chlorpyrifos (the chlorpyrifos concentration range stated by the company is 0.01–0.001 μg/g, but the exact concentration for each sample is unknown) was supplied by Pribolab Bioengineering Co., Ltd. (Qingdao, China). The proposed method and the GC-MS method were employed to determine the chlorpyrifos content in tea samples. Table 2 displays the detection results. Furthermore, two-tailed paired *t*-tests between the detection results of the MC-UVE, CARS, IRIV, and VISSA models and those of the GC-MS method were performed. The generated results were as follows: *p*-value (MC-UVE vs. GC-MS) = 0.24, *p*-value (CARS vs. GC-MS) = 0.27, *p*-value (IRIV vs. GC-MS) = 0.41, and *p*-value (VISSA vs. GC-MS) = 0.45. The results implied no significant difference between the proposed method and the GC-MS method regarding prediction performance (*p*-value > 0.05).

## 4. Conclusions

This study proposed a strategy combining SERS and intelligent variable selection models to determine chlorpyrifos residue in tea samples. The synthesized Au NSs guaranteed the successful acquisition of the SERS spectra of chlorpyrifos-spiked tea extracts. Four intelligent variable selection models, MC-UVE, CARS, IRIV, and VISSA, were executed on the preprocessed SERS spectra data, and the results revealed that these models effectively predicted chlorpyrifos residue concentration in tea samples. Specifically, the VISSA model showed superior prediction performance than the other models, with RMSEC = 0.1135, $R_{c}^{2}$ = 0.9968, RMSEP = 0.1580, $R_{p}^{2}$ = 0.9892, and RPD = 9.6246. The excellent stability of the proposed method was validated according to two aspects: (1) the fabricated Au NSs showed excellent repeatability and reproducibility; and (2) the developed intelligent variable selection models demonstrated outstanding stability. The high sensitivity of the proposed method was assessed by estimating LOD values, and the LOD values of the various models were all less than the MRL of chlorpyrifos in tea set by the European Union (1.0 × 10^−2^ μg/g). Additionally, two-tailed paired *t*-tests on the detection results of the MC-UVE, CARS, IRIV, and VISSA models and those of the GC-MS method implied no significant difference between the proposed method and the GC-MS method regarding prediction performance (*p*-value > 0.05). In conclusion, the proposed method is promising for quantitatively determining chlorpyrifos residue in tea.

## Figures and Tables

**Table 1:** Prediction results of chlorpyrifos content in tea samples by various models.

	nLVs ^a^	nWAV ^b^	Calibration Set		Prediction Set		
			RMSEC	$R_{c}^{2}$	RMSEP	$R_{p}^{2}$	RPD
PLS	8	534	0.1475	0.9925	0.3015	0.9513	4.5310
MC-UVE	10	100	0.1420	0.9932	0.2342	0.9712	5.8928
CARS	9	23	0.1315	0.9944	0.2117	0.9745	6.2617
IRIV	7	27	0.1246	0.9956	0.1998	0.9796	7.0028
VISSA	10	78	0.1135	0.9968	0.1580	0.9892	9.6246

nLVs ^a^: the optimal number of latent variables. nWAV ^b^: the number of selected wavelengths.

**Table 2:** Detection results (μg/g, Mean ^a^ ± SD ^b^) of chlorpyrifos content in tea samples by the proposed and GC-MS methods.

	Proposed Method				GC-MS
	MC-UVE	CARS	IRIV	VISSA	
Sample 1	0.00975 ± 6.4 × 10^−4^	0.00971 ± 7.1 × 10^−4^	0.00983 ± 2.5 × 10^−4^	0.00985 ± 2.6 × 10^−4^	0.00990 ± 1.1 × 10^−4^
Sample 2	0.00278 ± 7.1 × 10^−4^	0.00282 ± 8.2 × 10^−4^	0.00294 ± 3.7 × 10^−4^	0.00305 ± 3.5 × 10^−4^	0.00307 ± 0.8 × 10^−4^
Sample 3	0.00546 ± 7.5 × 10^−4^	0.00549 ± 6.9 × 10^−4^	0.00561 ± 3.9 × 10^−4^	0.00572 ± 4.4 × 10^−4^	0.00587 ± 1.4 × 10^−4^
Sample 4	0.00958 ± 6.8 × 10^−4^	0.00957 ± 9.5 × 10^−4^	0.00966 ± 4.5 × 10^−4^	0.00981 ± 4.3 × 10^−4^	0.00972 ± 1.7 × 10^−4^
Sample 5	0.00485 ± 6.6 × 10^−4^	0.00494 ± 5.3 × 10^−4^	0.00501 ± 4.1 × 10^−4^	0.00508 ± 1.7 × 10^−4^	0.00529 ± 0.6 × 10^−4^

Mean ^a^: average value of 10 tests. SD ^b^: standard deviation of 10 tests.

## Data Availability

The original contributions presented in the study are included in the article/Supplementary Materials, further inquiries can be directed to the corresponding author.

## Supplementary Materials

The following supporting information can be downloaded at: https://www.mdpi.com/article/10.3390/foods13152363/s1 [47,48,49], Figure S1: (A) The process of the synthesis of Au NPs. (B) The process of the synthesis of Au NSs; Figure S2: (A) The process of generating binary matrix. (B) The performance of the inclusion and exclusion of one variable is compared; Figure S3: The processes of binary matrix sampling (BMS) and weighted binary matrix sampling (WBMS). Binary matrix 2 is used for sampling, and each row of the matrix is a run of sampling where the number “1” represents the variable that is selected for modeling while the number “0” represents the variable that is not selected; Figure S4: The TEM image of Au NPs; Figure S5: The change in weights of each wavelength during the iteration in VISSA; Figure S6: (a) The collected SERS spectrum of chlorpyrifos solution (concentration: 100 μg/mL) based on Au NSs, and (b) the collected Raman spectrum of chlorpyrifos solution (concentration: 1.0 × 10^6^ μg/mL).
