# Supplementary material for: A Sensitive SERS Sensor Combined with Intelligent Variable Selection Models for Detecting Chlorpyrifos Residue in Tea
Source: Foods. 2024 Jul 26;13(15):2363. doi: 10.3390/foods13152363 (PMC11311742; doi:10.3390/foods13152363)
Supplement: Supplementary file 1 [file foods-13-02363-s001.zip › foods-3085765-supplementary.pdf]

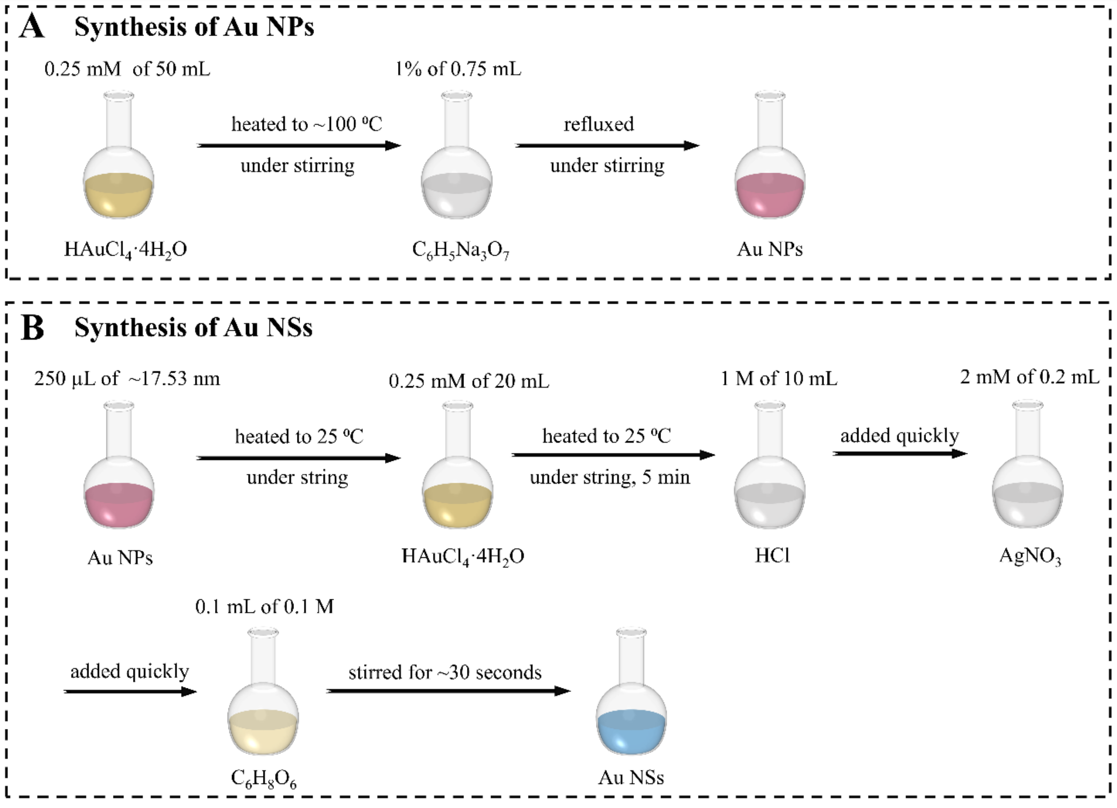

Figure S1. (A) The process of the synthesis of Au NPs. (B) The process of the synthesis of Au NSs.

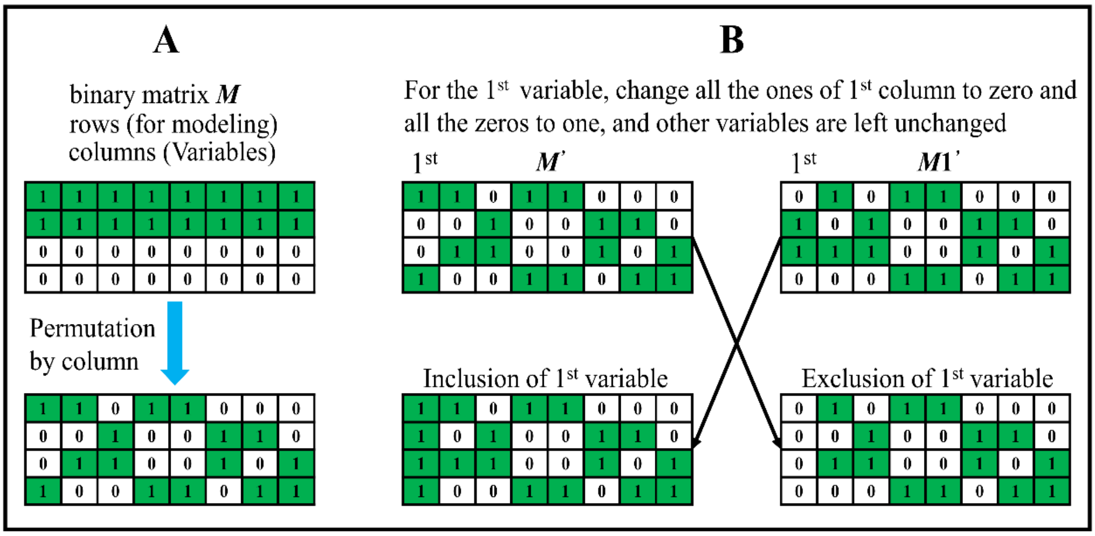

Figure S2. (A) The process of generating binary matrix. (B) The performance of the inclusion and exclusion of one variable is compared.

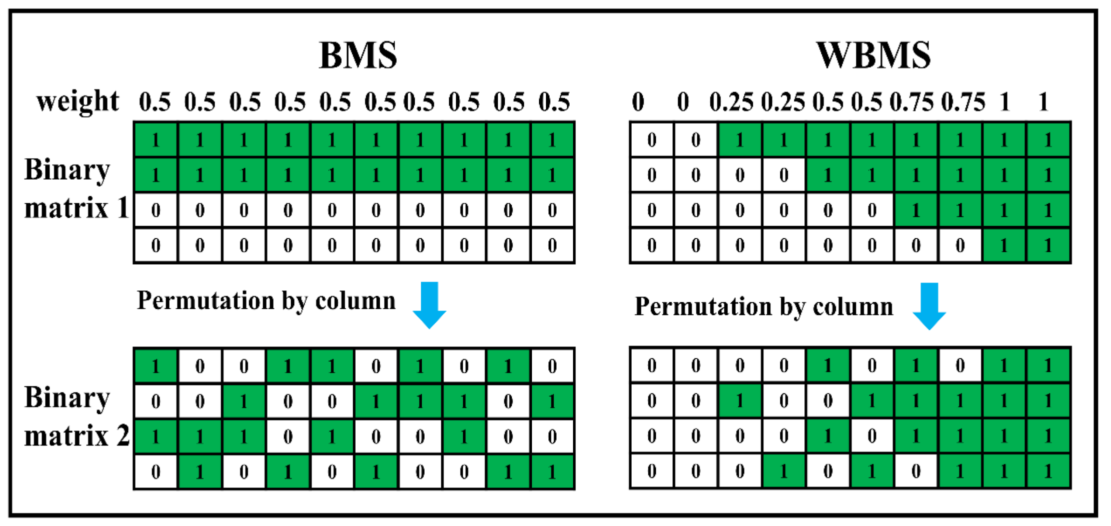

Figure S3. The processes of binary matrix sampling (BMS) and weighted binary matrix sampling (WBMS). Binary matrix 2 is used for sampling, and each row of the matrix is a run of sampling where the number “1” represents the variable that is selected for modeling while the number “0” represents the variable that is not selected.

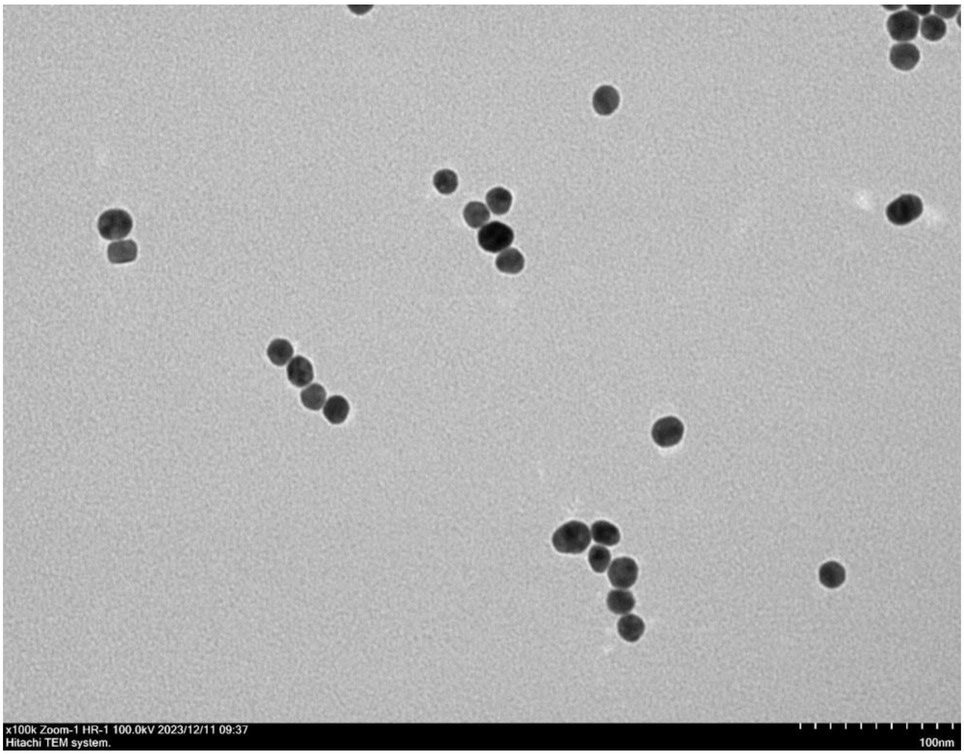

Figure S4. The TEM image of Au NPs.

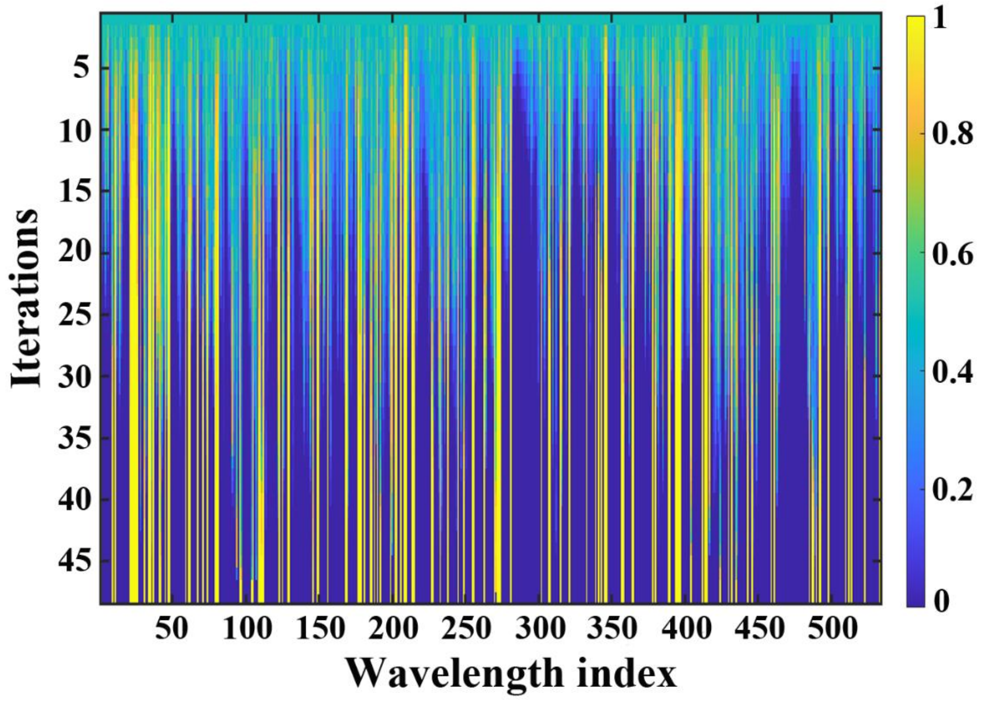

Figure S5. The change of weights of each wavelength during the iteration in VISSA.

Mean Relative Error Evolution Approach

Oleneva *et al.* proposed a LOD estimation approach based on the idea of visual representation of mean relative error (MRE) increment evolution dependent on an analyte concentration [1]. Basically, MRE can be calculated by the formula:

$$MRE = \frac{|M-P|}{M} \qquad (1)$$

where *M* is the measured value (taken as actual), and *P* represents the predicted value. MRE describes the difference between the measured analyte concentration and the predicted one, related to the actual value to avoid the effect of different concentration magnitude on the borders of the concentration range. Obviously, if the analyte concentrations are lower than the LOD, average MRE must be high, because an instrument cannot properly determine such a small amount of the analyte. The idea is to find the analyte concentration, starting from which the average MRE value significantly decreases, according to the following procedure:

(1) sort the column with measured analyte concentration y_n in ascending order ($y_{n\ meas}$ becomes $\tilde{y}_{n\ meas}$);
(2) calculate MRE for each sample;
(3) average MRE values and the measured concentrations for the first n samples (with the lowest concentration) by using the following formula step by step:

$$MRE_{n'} = \frac{1}{n'}\sum_{n=2}^{n'} \frac{|\tilde{y}_{n\ meas} - \tilde{y}_{n\ pred}|}{\tilde{y}_{n\ meas}} \quad n' = 2,3,\ldots,N \qquad (2)$$

where $\tilde{y}_{n\ pred}$ represents the predicted concentrations.

(4) calculate the increment of averaged MRE by the following formula:

$$\Delta\langle MRE\rangle = |MRE_{n+1'} - MRE_{n'}| \qquad (3)$$

Finally, a LOD for the given analyte is a concentration, starting from which the $\Delta\langle MRE\rangle$ value fluctuations around zero are equal or less than 1%.
The Matlab code for LOD estimation algorithm can be available online at http:// www.mdpi.com/1424-8220/19/6/1359/s1.

Analysis of chlorpyrifos residue in tea samples by GC-MS

The following sample preparation steps should be carried out before GC-MS (GCMS-QP2010, Shimazdu, Japan) analysis [2]. Weight 5 g of ground tea samples (ground by a A11 pulverizer) into a 50 mL plastic centrifuge tube to which 10 mL water and 30 mL ethyl acetate were added for extraction. The mixture was homogenized for 2min and centrifugated at 5000 rpm for 5min. After that, the supernatant was collected into a 250 mL round-bottom flask with anhydrous sodium sulfate. The residue was extracted again with 30 mL of ethyl acetate, and the supernatant was dehydrated with anhydrous sodium sulfate. The mixture of two extracts was condensed to near dryness by a rotary evaporator in a 40 ºC water bath. Subsequently, the dry residue was dissolved with 2 mL of ethyl acetate/n-hexane (1:1, v/v) in a round-bottom flask, and the resultant was then subjected to solid phase extraction (SPE).
The active carbon SPE column (ENVI-Carb) was connected to the florisil SPE column (SIMON Florisil). The coupled SPE columns were rinsed with 6 mL of ethyl acetate/n-hexane (1:1, v/v) in advance. The 2 mL of extract in the round-bottom flask was injected into the coupled SPE columns and eluted with an additional 6 mL of ethyl acetate/n-hexane (1:1, v/v). The eluate was evaporated to near dryness in a 40 °C water bath. The residue was dissolved with 1.0 mL of ethyl acetate and transferred into a 2 mL vial for GS-MS analysis. The GC-MS system had an automatic sampler (AOC-20i + s), automatic injector, and fused silica capillary column: HP-5. The GC-MS solution software (release version 2.30) was used to obtain and analyze the data.

Calculating the enhancement factor (EF) of Au NSs

The EF value is calculated by the following equation [3]:

$$EF = \frac{I_{SERS} \times N_{bulk}}{N_{SERS} \times I_{bulk}} \quad (4)$$

where I_{SERS} and I_{bulk} are the intensities of the selected characteristic bands in the SERS and Raman spectrum, respectively; N_{SERS} and N_{bulk} are the numbers of probe molecules contributed to the SERS signal and bulk Raman signal, respectively. The formula for N can be expressed as:

$$N_{SERS} = N_A \times C_{\mathrm{SERS}} \times V_{SERS} \times \frac{S_{Laser}}{S_{SERS}} \quad (5)$$

$$N_{bulk} = N_A \times C_{bulk} \times V_{bulk} \times \frac{S_{Laser}}{S_{bulk}} \quad (6)$$

where N_A is Avogadro constant. For SERS measurement, a certain volume V_{SERS} of Au NSs with chlorpyrifos solution is dispersed on an area of S_{SERS} at a concentration of C_{SERS}. For Raman measurement, a certain volume V_{bullk} of chlorpyrifos solution is dispersed on an area of S_{bulk} at a concentration of C_{bulk}. The V_{SERS} and V_{bullk} are all 5

μL, thus the S_{SERS} and S_{bulk} are estimated to be the same. Hence, the Eq. (4) can be written as:

$$EF = \frac{I_{SERS} \times C_{bulk}}{I_{bulk} \times C_{SERS}} \quad (7)$$

Herein three characteristic band of chlorpyrifos at 1143, 1266, and 1447 cm^{-1} are selected, the corresponding I_{SERS} values are 1745.94(a.u.), 2732.62(a.u.), and 1361.25(a.u.). The corresponding I_{bulk} values are 161.56(a.u.), 268.75(a.u.), and 125.12(a.u.). The C_{SERS} is 100 μg/mL, and the C_{bulk} is 1.0×10^6 μg/mL. Hence, the EF values of Au NSs are calculated to be 1.08×10^5, 1.02×10^5, and 1.09×10^5. Then, we take the mean of three EF values as the final EF = 1.06×10^5. Fig. S6 shows the collected SERS spectrum of chlorpyrifos solution (concentration: 100 μg/mL) based on Au NSs and the collected Raman spectrum of chlorpyrifos solution (concentration: 1.0×10^6 μg/mL).

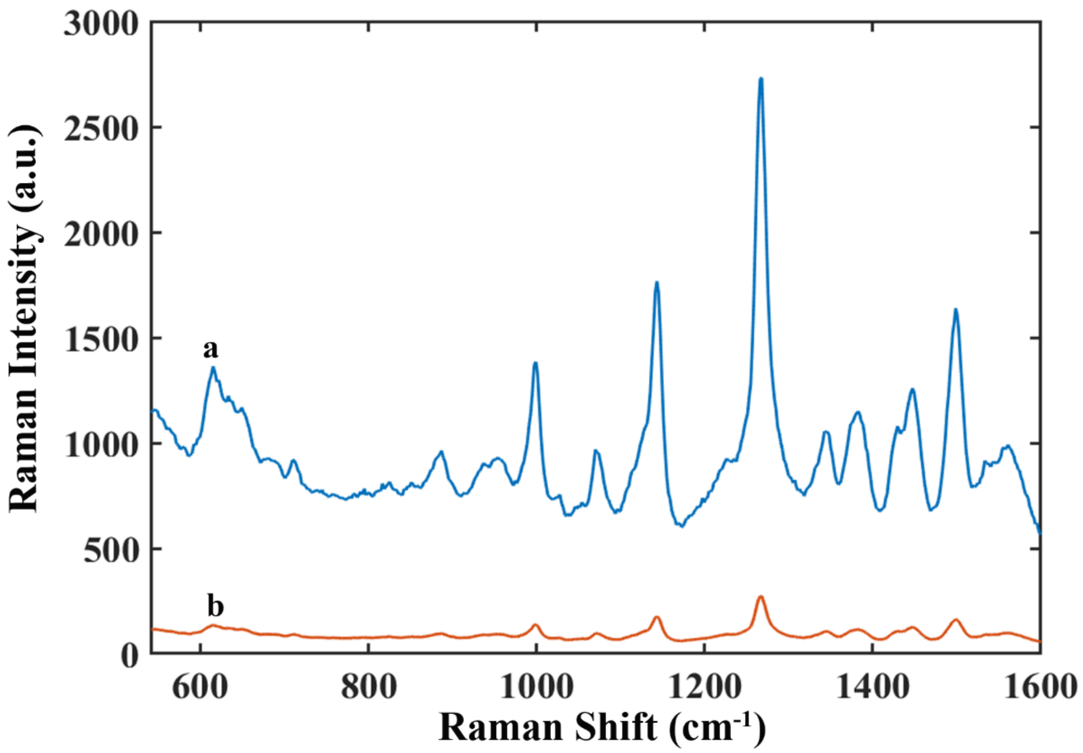

Fig. S6. (a) The collected SERS spectrum of chlorpyrifos solution (concentration: 100 μg/mL) based on Au NSs, and (b) the collected Raman spectrum of chlorpyrifos solution (concentration: 1.0×10^6 μg/mL).

Reference

[1] E. Oleneva, M. Khaydukova, J. Ashina, I. Yaroshenko, I. Jahatspanian, A. Legin, D. Kirsanov, A Simple Procedure to Assess Limit of Detection for Multisensor Systems, Sensors, 19 (2019): 1359.

[2] R.-Y. Hou, W.-T. Jiao, X.-S. Qian, X.-H. Wang, Y. Xiao, X.-C. Wan, Effective Extraction Method for Determination of Neonicotinoid Residues in Tea, Journal of Agricultural and Food Chemistry, 61 (2013): 12565-12571.

[3] X.Y. Ma, B.Y. Shao, Z.P. Wang, Gold@silver nanodumbbell based inter-nanogap aptasensor for the surface enhanced Raman spectroscopy determination of ochratoxin A, Analytica Chimica Acta, 1188 (2021): 339189.
